# Quality of High-Fibre Pasta Supplemented with Watermelon Rind Powder with Different Particle Sizes

**DOI:** 10.17113/ftb.62.01.24.8196

**Published:** 2024-03

**Authors:** Dien Quang Long, Thi Mien Trieu, Thi Thu Tra Tran, Nu Minh Nguyet Ton, Van Viet Man Le

**Affiliations:** 1 Department of Food Technology, Ho Chi Minh City University of Technology (HCMUT), 268 Ly Thuong Kiet Street, District 10, 700 000 Ho Chi Minh City, Vietnam; 2 Vietnam National University – Ho Chi Minh City (VNU-HCM), Linh Trung Ward, Thu Duc, 700 000 Ho Chi Minh City, Vietnam

**Keywords:** antioxidant activity, bioaccessibility, dietary fibre, pasta, watermelon rind

## Abstract

**Research background:**

Watermelon rind, a by-product of watermelon juice processing, contains large amounts of dietary fibre and phenols with antioxidant capacity. The use of agro-industrial by-products would both improve economic benefits and reduce environmental emissions. The aim of this research is to examine the effect of the particle size of watermelon rind powder on the quality of high-fibre pasta.

**Experiment approach:**

The nutritional, physical and physicochemical quality of three samples of watermelon rind powder, sieved through three sieves with aperture size of 400, 210 and 149 μm, were analysed. Durum wheat semolina with watermelon rind powder mass fraction of 10 % were mixed and used to make pasta. Nutritional, textural and cooking quality, sensory acceptability, *in vitro* glycaemic index and antioxidant bioaccessibility of high-fibre pasta with added watermelon rind powder of different particle sizes were evaluated and compared.

**Results and conclusions:**

When the sieve aperture size was reduced from 400 to 149 µm, the soluble dietary fibre and total phenolic contents of watermelon rind powder were increased by 35 and 15 %, respectively, while its insoluble dietary fibre content was decreased by 21 %. Decrease in sieve aperture size from 410 to 149 µm reduced phenolic bioaccessibility of the fortified pasta from 63 to 57 %, but enhanced its predicted glycaemic index from 50 to 69. It also decreased the pasta hardness by 13 %, but improved its elongation rate and tensile strength by 13 and 40 %, respectively. The finer the particles of the watermelon rind powder, the longer the optimal cooking time, the higher the water absorption index, and the lower the cooking loss of the supplemented pasta. Consumers did not notice any significant differences in the overall acceptability among all pasta samples.

**Novelty and scientific contribution:**

The particle size of the watermelon rind powder had a major effect on nutritional value, texture and cooking quality of the fortified pasta. In particular, the predicted glycaemic index and antioxidant bioaccessibility of high-fibre pasta were significantly affected by the particle size of the dietary fibre material used in the recipe.

## INTRODUCTION

The pasta has become a staple food for people around the world because it is cheap and easy to prepare (*1*). Pasta contains many carbohydrates, the main component of which is starch, while the fibre content is very low (0.9–1.9 g/100 g) (*1*). An adequate intake of dietary fibre (about 35 g/day for adults) has been linked to a reduction in many health concerns including digestive disorders, diabetes, colon cancer and coronary heart disease (*2*, *3*). Besides, pasta is poor in bioactive compounds such as antioxidants (*4*), which are responsible for reducing the risk of developing chronic diseases, including obesity, cardiovascular disease and various types of cancer, as well as ageing (*5*). In the last decade, functionalisation of pasta has attracted considerable attention (*6*). The common source of dietary fibre and antioxidants in the production of pasta is whole grain flour, which is ground from whole, unprocessed cereal grains such as wheat, oat, barley or rice (*7*). Other plant powders with a high fibre and antioxidant content such as fruit, vegetables (*4*), herb seeds (*1*) or flowers (*8*) are also added to the pasta recipe. Another way to increase the fibre and antioxidant content of pasta is to replace the dough water with fruit juice or fruit and vegetable puree (*4*). Over the last decade, agro-industrial by-products, particularly fruit by-products have been added to the pasta formulations to increase the dietary fibre and antioxidant content (*9*).

Watermelon, a member of the *Cucurbitaceae* family, is a valued fruit that grows in tropical and subtropical climates and it is known for its high nutritional value (*10*). In 2021, the productivity of watermelon was 101.6 million tonnes in the world and 1.5 million tonnes in Vietnam (*11*). The rind, which accounts for 30–41 % of the total mass of watermelon fruit, is main solid waste generated during watermelon juice processing (*10*). This by-product is usually used for the production of animal feed or as fertiliser. However, watermelon rind contains large amounts of total dietary fibre, proteins and minerals (*10*). Watermelon rind is also reported to have high antioxidant activity due to the presence of phenols, l‐citrulline, terpenoids, saponins and alkaloids (*12*). Among them, phenolic compounds, especially 4‐hydroxybenzoic acid and vanillin, are the main antioxidants of watermelon rind (*10*, *12*, *13*). Various bioactive substances such as citrulline, pectin and polysaccharides with bioactivity are extracted from watermelon rind (*13*). In addition, the direct use of watermelon rind in the formulation of different food products such as cookies, bread and noodles (*13*) is reported to increase their dietary fibre and phenolic content.

The texture profile of pasta is influenced by the particle size of the flour (*9*). In addition, the particle size of raw materials also affects the bioaccessibility of nutrients and antioxidants in food products (*14*). Nevertheless, the effects of particle size of watermelon rind powder on the quality of high-fibre pasta have not been reported in the literature. The aim of this study is to evaluate the effects of particle size of watermelon rind powder on nutritional, textural and cooking quality, and overall acceptability of high-fibre pasta. In addition, the glycaemic index and bioaccessibility of phenolic compounds of the obtained product samples were evaluated by an *in vitro* test.

## MATERIALS AND METHODS

### Materials

Watermelon (*Citrullus lanatus* (Thunb.) Matsum. & Nakai) fruits were collected from a local farm (Long An province, Vietnam). At the laboratory, the fruit exocarp was separated and the outer green peel was manually removed. The white rind was cut into 2 cm×5 cm×0.2 cm pieces, dried at 50 °C to achieve 12 % moisture content. About 200 g dried watermelon rind were placed in a hammer mill (Binh Minh Ltd., Ha Noi, Vietnam) with eighteen swinging hammers attached to the rotor and each batch was rotated at 2400 rpm for 2 min. The obtained watermelon rind powder from all milling batches was mixed well and divided into three parts. Each part was sieved through a sieve. In this study, 40-, 70- and 100-mesh sieves with apertures of 400, 210 and 149 μm, respectively, were used. The obtained watermelon rind powder fractions were vacuum-packed in polyethylene bags, stored at 4 °C and used for experiments within 4 weeks. Durum wheat semolina was provided by Vietnam Wheat Milling Co., Ltd. (Ba Ria–Vung Tau Province, Vietnam). Sodium chloride was procured from Vietnam Southern Salt Group (Ho Chi Minh City, Vietnam).

Enzyme preparations including Termamyl® SC DS with α-amylase activity, Dextrozyme® GA with glucoamylase activity and Alcalase® 2.5 L with protease activity were purchased from Novozymes (Bagsværd, Denmark) and used for fibre determination. Salivary α-amylase, pepsin from porcine gastric mucosa, pancreatin from porcine pancreas (8×USP specifications) and bile salts were bought from Merck KGaA (Darmstadt, Germany) and used for *in vitro* experiments. Chemicals of analytical grade including Folin-Ciocalteu reagent, 2,2-diphenyl-1-picrylhydrazyl (DPPH), gallic acid (GA), Trolox, 2,4,6-Tri(2-pyridyl)-s-triazine (TPTZ) and 3,5-dinitrosalicylic acid (DNS) were provided from Merck KGaA.

### Pasta making in the laboratory

Four pasta samples were prepared: control sample P (without the addition of watermelon rind) and three samples with added watermelon rind: P400, P210 and P149 in which 10 % durum wheat semolina was replaced by watermelon rind powder samples sifted through a 400-, 210- and 149-μm aperture sieve, respectively. The pasta was prepared according to Nguyen *et al.* (*3*) with slight modifications. About 150 g mixture of durum semolina with watermelon rind powder and table salt (0.5 % on the blend dry mass) were mixed in a dough mixer (model 5K5SS-Heavy Duty KitchenAid; Whirlpool Co., Benton Harbor, MI, USA) for 5 min using a flat beater. About 70 mL distilled water at 42 °C was then added and mixed at 120 rpm for 2 min. The flat beater was replaced with a dough hook and the mixture was kneaded at 120 rpm for another 20 min to make a pasta dough with 39 % moisture content. The obtained dough was then fed to an extruder (model HR2365/05; Philips Co., Eindhoven, the Netherlands) with die size of 4 mm×2 mm and extrusion pressure of 70.6 MPa. The extruded pasta strands were dried at 50 °C for 5 h in a convective dryer. The dried pasta was kept in polyethylene pouches at 4 °C until analysis.

### Nutritional quality

Moisture content was determined by drying at 105 °C to constant mass using a moisture analyzer (model ML-50; A&D Co., Tokyo, Japan). Total ash was estimated by incineration at 600 °C using a muffle furnace (model EF11/8B-Lenton Furnaces, Carbolite Gero Ltd, Sheffield, UK), following the AOAC method 942.05 (*15*). Total protein was determined by Kjeldahl digestion using AOAC method 979.09 (*16*) and the protein-nitrogen conversion factor of 5.7. Total lipids were measured by Soxhlet extraction following AOAC method 930.09 (*17*). Starch was determined using AOAC method 996.11 (*18*). Reducing sugars were evaluated by a spectrophotometric method using DNS reagent (*19*). Total sugar was assessed by AOAC method 945.66 (*20*) using Fehling reagent. Insoluble dietary fibre (IDF), soluble dietary fibre (SDF) and total dietary fibre (TDF) were measured by enzymatic-gravimetric principle following AOAC methods 991.42 (*21*), 993.19 (*22*) and 991.43 (*23*), respectively. Pectin was determined by extraction and subsequent precipitation with ethanol according to the procedure reported by Petkowicz *et al.* (*24*). About 10 g sample were added to absolute ethanol and the mixture was boiled under reflux for 20 min. An alcohol-insoluble residue was collected by filtrating, washing with 20 mL absolute ethanol and drying. Pectins were extracted from this residue using the boiling 0.1 M HNO_3_ solvent under reflux for 1 h (*m*(residue):*V*(HNO_3_)=1:25). The extract was isolated by centrifugation (model 3K30; Sigma Zentrifugen Ltd., Osterode am Harz, Germany) at 15 400×*g* for 20 min and treated with ethanol (2 volumes) for 16 h at 4 °C. The precipitated pectins were then separated, washed thrice with 10 mL ethanol and dried under vacuum.

### Phenolic compounds and antioxidant activity

Phenols were extracted with *φ*(ethanol)=60 % under the extraction conditions described elsewhere (*3*). In 15-mL centrifugal tubes, each ground sample (1 g) and 10 mL of solvent were added. After shaking the tubes for 2 h at room temperature, they were centrifuged (model 3K30; Sigma Zentrifugen Ltd.) at 3000×*g* at 4 °C for 15 min. The supernatants were kept out of the light and stored at −18 °C for a week. The extract was used to evaluate total phenolic content (TPC), total flavonoid content (TFC) and total anthocyanin content (TAC) as well as antioxidant activity.

Total phenols were determined using Folin-Ciocalteu reagent and spectrophotometric method (*3*) and expressed in mg gallic acid equivalents (GAE) per kg dry mass. About 0.2 mL extract was mixed with 1.5 mL 10-fold diluted Folin-Ciocalteu reagent, vortexed for 30 s and equilibrated for 5 min at room temperature. Then, 1.5 mL Na_2_CO_3_ (*γ*=60 g/L) was added and vortexed for 30 s. The reaction mixture was kept in the dark at room temperature for 90 min, and absorbance at 725 nm was recorded against a solvent blank.

Total flavonoids were determined spectrophotometrically using the procedure reported previously (*25*) and expressed in mg quercetin equivalents (QE) per kg dry mass. Briefly, 250 μL of extract were mixed with 2720 μL distilled water and 120 μL 0.5 mol/L NaNO_2_ for 5 min. A volume of 120 μL AlCl_3_·6H_2_O (*γ*=10 g/100 mL) were added, vortexed and left to react for another 5 min. Finally, 800 μL NaOH (*c*=1 M) were added, left for 5 min and then the absorbance was measured at 510 nm.

Total anthocyanins were measured by pH differential method and expressed in mg cyanidin-3-glucoside equivalent (CGE) per kg dry mass (*25*). For quantification, standard solutions of 0.025 M KCl (pH=1.0) and 0.4 M CH_3_COONa (pH=4.5) were prepared. About 400 μL of extract were added to two tubes, each containing 3.6 mL of the respective standard solution. Each sample was measured at 2 wavelengths (λ=520 and 700 nm) using a spectrophotometer with a 1 cm optical path cuvette.

Fe(III) reducing antioxidant power (FRAP) and DPPH radical scavenging activity were determined according to the procedures described elsewhere (*3*) and expressed in µmol Trolox equivalents (TE) per kg dry mass of the sample. For DPPH assay, 0.1 mL extract was mixed with 3.9 mL 60 μM DPPH solution in methanol. After incubation in the dark at ambient temperature, absorbance at 515 nm was measured at the beginning and after 30 min against a solvent blank. For FRAP assay, 25 mL of 0.3 M acetate buffer (pH=3.6), 2.5 mL 10 mM TPTZ solution in 40 mM HCl and 2.5 mL of 20 mM FeCl_3_·6H_2_O were mixed. About 0.2 mL of the diluted extract was added subsequently. After incubation at 37 °C for 5 min in the dark, absorbance was measured at 593 nm against a solvent blank.

### Physical properties

The particle size of durum wheat semolina and watermelon rind powder was measured using a laser particle size analyzer (model MAZ3000; Malvern Instruments Ltd, Malvern, UK). The results were expressed as particle size range, mean value (*D*_[4,3]_), span value and specific surface area (*A*_m_) (*3*). Bulk density was measured according to a procedure described elsewhere (*26*).

Water holding capacity (WHC) was determined according to the procedure described by Mai *et al.* (*2*). Water solubility index (WSI) and swelling capacity were analysed according to the procedure reported by Ming *et al.* (*26*).

Instrumental colour was measured using a chromameter (model CCM-3700A; Konica Minolta Co., Osaka, Japan) and expressed as L* (lightness), a* (redness) and b* (yellowness) values. Total colour difference (Δ*E*) was calculated by a previously described formula (*3*).

### Cooking quality

The cooking properties of pasta including optimal cooking time (OCT), cooking loss (CL), water absorption index (WAI) and swelling index (SI) were determined following the procedure reported by Nguyen *et al.* (*3*).

### Textural properties of cooked pasta

Textural properties of cooked pasta were evaluated by a texture analyzer (TA.XTplusC; Stable Micro Systems Co., Godalming, UK) with Exponent Connect Lite v. 6.1.7 software (*27*). Hardness, adhesiveness, tensile strength (MPa) and elongation rate (%) were measured according to Nguyen *et al.* (*3*).

### In vitro digestion of cooked pasta

The *in vitro* digestion of cooked pasta was determined according to the procedure by Lucas-González *et al.* (*28*). In brief, fluids with electrolyte stock, enzymes, CaCl_2_ and water were prepared, and the pH was adjusted to the required value using 1 M HCl or NaOH solutions. The digestion occurred in 50-mL Falcon tubes in a shaking bath at 37 °C and 30 rpm for required durations. Before digestion, pasta samples were cooked to their OCT, cooled for 10 min and manually cut into 2.0–5.0 mm pieces. About 5 g of cooked pasta were mixed with 5 mL of simulated salivary fluid for a 2-minute incubation. Then, 10 mL of simulated gastric fluid were added and after adjusting the pH to 2, the gastric phase ran for 2 h. Next, 20 mL of simulated intestinal fluid were added, adjusting the pH to 7, which required an additional 2-hour incubation for the intestinal phase. Blank reagents were used for each simulated phase. At the end of digestion, samples were centrifuged (model 3K30; Sigma Zentrifugen Ltd.) at

12 000×*g* at 4 °C for 10 min to separate the pellet and soluble fractions. The soluble fraction was collected, weighted and extracted three times with 2.5 mL of of *φ*=50 % aqueous solvent for 8 h at room temperature. After centrifugation (model 3K30; Sigma Zentrifugen Ltd.) at 127×*g* for 5 min, the supernatant was filtered through a 0.45-µm syringe for analysis. Bioaccessibility index of total phenols (%) was calculated by the following formula (*28*):


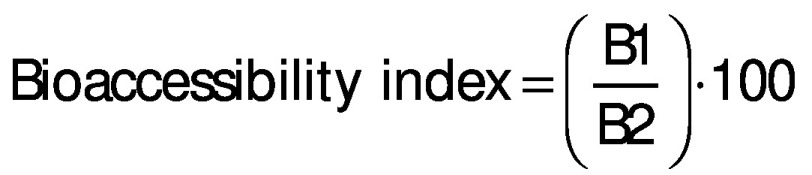
 /1/

where B1 is the TPC (mg) of the total volume of the obtained supernatant and B2 is the TPC (mg) of 5 g cooked pasta sample used in the *in vitro* digestion.

The *in vitro* glycaemic index for all pasta samples was determined following the procedure reported by Mai *et al.* (*29*). White bread was used as a reference sample (its glycaemic index was 71). The method principle is based on the evaluation of reducing sugar content released during the digestion. Sample was hydrolyzed with enzymes that simulated the human digestive system (pepsin, pancreatin and amyloglucosidase) under the appropriate reaction conditions. After every 15 min of incubation (from 0 to 180 min), a portion of the aliquot was collected, enzymatically inactivated, centrifuged (model 3K30; Sigma Zentrifugen Ltd.) and the obtained supernatant was used to measure reducing sugars spectrophotometrically, with DNS reagent and glucose as a standard. Predicted glyceamic index was calculated using the formula described by Mai *et al.* (*29*).

### Overall acceptability

Sixty non-smokers and untrained panellists (40 men and 20 women) aged from 18 to 28 were selected among the students and staff of Ho Chi Minh City University of Technology (Vietnam). They were recruited because they consumed pasta frequently (at least once a week). All pasta samples were assigned 3-digit codes and served one at a time in a randomized sequence. Water was supplied between samples for mouth cleansing. A 9-point hedonic scale ranging from point 1 (extremely dislike) to point 9 (extremely like) was used to evaluate overall acceptability.

### Statistical analysis

Each pasta sample was prepared in triplicate. The results were shown as mean value±standard deviation. One-way analysis of variance and Tukey's *post-hoc* test (significance level set at p<0.05) were applied to distinguish the significant difference between mean values using Statgraphics Centurion program, v. 18.1.12 (*30*).

## RESULTS AND DISCUSSION

### Nutritional quality, antioxidant activity and physical properties of watermelon rind powder and wheat semolina

Table 1 shows nutritional quality, antioxidant activity and physical properties of watermelon rind powder and semolina used in this study. When the sieves with smaller apertures were used, the obtained watermelon rind powder sample had a narrower range of particle size distribution, its mean particle size was smaller, while its specific surface area and bulk density were larger, and the uniformity of particle size was significantly improved. The particle size distribution of durum wheat semolina was considerably wider than that of all watermelon rind powder samples. The finer the particles of fibre material, the better the dispersion in the food system (*31*).

**Table 1:** Nutritional quality, antioxidant activity and physical properties on dry mass basis of watermelon rind powder and durum wheat semolina

Parameter	Watermelon rind powder *d*(aperture sieve)/μm			Durum wheat semolina
400	210	149
*d*(particle)/μm	4–1849	3–352	2–310	2–3080
*D*_[4,3]_/μm	(148±7)ᶜ	(85±4)ᵇ	(70±3)ᵃ	(183±6)ᵈ
*A*_m_/(m^2^/kg)	(271.5±5.1)ᵃ	(354.0±5.3)ᵇ	(379.5±9.5)ᶜ	(277±14)ᵃ
SPAN	(3.2±0.1)ᵈ	(2.4±0.1)ᵇ	(2.1±0.1)ᵃ	(2.9±0.1)ᶜ
*ρ*/(g/cm^3^)	(0.351±0.004)ᵃ	(0.405±0.01)ᵇ	(0.44±0.02)ᶜ	(0.83±0.01)ᵈ
*w*(water)/%	(7.8±0.4)ᵃ	(7.9±0.2)ᵃ	(8.2±0.4)ᵃ	(12.4±0.1)ᵇ
*w*(TDF)/(g/100 g)	(41.7±0.4)ᵈ	(40.4±0.8)ᶜ	(38.6±0.6)ᵇ	(3.6±0.0)ᵃ
*w*(IDF)/(g/100 g)	(29.6±0.8)ᵈ	(27.0±0.9)ᶜ	(23.5±0.9)ᵇ	(2.7±0.1)ᵃ
*w*(SDF)/(g/100 g)	(12.2±0.3)ᵇ	(13.4±0.2)ᶜ	(15.1±0.5)ᵈ	(0.8±0.0)ᵃ
*w*(pectin)/(g/100 g)	(7.5±0.5)ᵃ	(8.2±0.4)ᵇ	(10.1±0.5)ᶜ	n.d.
*ζ*(IDF/SDF)/(g/g)	(2.4±0.1)ᶜ	(2.0±0.1)ᵇ	(1.6±0.1)ᵃ	(3.4±0.2)ᵈ
*w*(starch)/(g/100 g)	(2.5±0.1)ᵃ	(2.4±0.1)ᵃ	(2.4±0.1)ᵃ	(70.7±1.4)ᵇ
*w*(total sugar)/(g/100 g)	(9.5±0.3)ᵇ	(10.9±0.4)ᶜ	(11.9±0.3)ᵈ	(3.7±0.0)ᵃ
*w*(reducing sugar)/(g/100 g)	(7.5±0.2)ᵇ	(8.3±0.5)ᶜ	(9.3±0.1)ᵈ	(0.7±0.0)ᵃ
*w*(protein)/(g/100 g)	(13.0±0.3)ᵃ	(13.6±0.2)ᵇ	(15.1±0.5)ᶜ	(13.3±0.4)ᵃᵇ
*w*(lipid)/(g/100 g)	(2.9±0.1)ᵈ	(2.6±0.1)ᶜ	(2.3±0.1)ᵇ	(1.8±0.1)ᵃ
*w*(ash)/(g/100g)	(12.8±0.1)ᵈ	(12.2±0.2)ᶜ	(11.1±0.3)ᵇ	(0.5±0.2)ᵃ
Total phenolics as *w*(GAE)/(mg/kg)	(7128±152)ᵇ	(7716±310)ᶜ	(8193±88)ᵈ	(269±7)ᵃ
Total flavonoids as *w*(QE)/(mg/kg)	(4433±11)ᵃ	(4539±18)ᵇ	(4720±17)ᶜ	n.d.
Total anthocyanin as *w*(CGE)/(mg/kg)	(393±19)ᶜ	(355±17)ᵇ	(315±13)ᵃ	n.d.
DPPH radical scavenging activity as *b*(TE)/(μmol/kg)	(27186±1295)ᵇ	(32384±1240)ᶜ	(34820±932)ᵈ	(2499±58)ᵃ
Fe(III) reducing power as *b*(TE)/(μmol/kg)	(11862±467)ᵇ	(18223±807)ᶜ	(21141±947)ᵈ	(1148±14)ᵃ
L*	(81.0±0.1)ᵃ	(81.1±0.0)ᵃ	(81.6±0.3)ᵇ	(91.2±0.0)ᶜ
a*	(2.3±0.0)ᵇ	(2.7±0.0)ᶜ	(2.7±0.0)ᶜ	(0.9±0.0)ᵃ
b*	(19.6±0.1)ᵇ	(20.3±0.0)ᶜ	(20.8±0.1)ᵈ	(10.0±0.0)ᵃ
WHC/(g/g)	(5.9±0.1)ᵈ	(5.1±0.0)ᶜ	(4.7±0.1)ᵇ	(0.9±0.0)ᵃ
SC/(mL/g)	(10.4±0.2)ᵈ	(7.0±0.3)ᶜ	(5.9±0.1)ᵇ	(1.0±0.0)ᵃ
WSI/%	(42.2±0.2)ᵇ	(44.9±0.3)ᶜ	(47.3±0.8)ᵈ	(13.1±0.5)ᵃ

Values with different lowercase letters in the same row are significantly different (p<0.05). n.d.=not detected, TDF=total dietary fibre, IDF=insoluble dietary fibre, SDF=soluble dietary fibre, GAE=gallic acid equivalent, QE=quercetin equivalent, CGE=cyanidin-3-glucoside equivalent, DPPH=2,2-diphenyl-1-picrylhydrazyl, TE=Trolox equivalent, WHC=water-holding capacity, SC=swelling capacity, WSI=water solubility index

The TDF content of watermelon rind powder samples was much higher than that of durum wheat semolina. The exocarp of watermelon fruit contains dense fibrous tissue (*32*) to protect the flesh. Fibre components identified in the watermelon rind were cellulose, hemicellulose, lignin and pectin, with pectin being a predominant soluble fibre (*13*). When the sieve pore size was reduced from 400 to 149 µm, the TDF and IDF contents of watermelon rind powder were decreased by 7 and 21 %, respectively, while its SDF and pectin contents were increased by 24 and 35 %, respectively. It is reported that the fibre components of plant materials are hard to grind into fine particles because of their high surface energy (*33*, *34*). As a result, the large particles of the ground material contained more dietary fibre than the small ones. Similar change in the dietary fibre content of pulverized potato peels was also reported when the mean particle size was reduced (*35*). Reducing the aperture size of the sieve from 400 to 149 µm resulted in a reduced IDF/SDF ratio of watermelon rind powder, but its value was always lower than that of durum wheat semolina. In addition, the starch content of the three watermelon rind powder samples was not significantly different. In contrast, the durum wheat semolina contained substantially more starch than the watermelon rind powder. Decreasing the mean particle size of watermelon rind powder increased the total sugar, reducing sugar and protein content. It is reported that proteins and sugars are entrapped in the fibre matrix of watermelon rind (*13*) and could be released during milling. The durum wheat semolina contained less total and reducing sugar than the watermelon rind, while its protein content was similar to or slightly lower than that of watermelon rind. When the sieve opening size was reduced from 400 to 149 µm, the lipid and ash content of watermelon rind powder decreased moderately. The ash content of watermelon rind was 22.8−25.6 times higher than that of durum wheat semolina. A previous study has shown that watermelon rind contains various minerals such as potassium, calcium, magnesium and iron, which are essential for human nutrition (*13*). Watermelon rind was therefore a source of dietary fibre and different nutrients for the development of high-fibre foods.

The antioxidant content and activity of watermelon rind powder were much higher than those of semolina. The smaller the aperture size of the sieve, the higher the TPC and TFC of the watermelon rind powder. According to Bender *et al.* (*36*), fine grinding could break down the bonds between phenolics and fibre, resulting in a better extraction of phenols from plant materials. An increase in the TPC was also observed with a decrease in mean particle size of peel powder from pomegranate (*37*) and apple fruits (*38*). However, the TAC of watermelon rind powder decreased marginally when the sieve opening size was reduced. When the sieve aperture size was reduced from 400 to 149 µm, the antioxidant capacity of watermelon rind powder measured by DPPH and FRAP assays increased by 28 and 78 %, respectively. Among the bioactive compounds quantified in the study, the total phenols had the highest correlation with the antioxidant activity (r=0.989 for DPPH assay and r=0.989 for FRAP assay). There was a significant positive correlation between the antioxidant activity measured by DPPH and FRAP assays (r=1.000), indicating that the antioxidant compounds in watermelon rind simultaneously showed antioxidant capacity by both mechanisms: DPPH radical scavenging and electron transfer (*39*).

The difference in the colour values of the three watermelon rind powder samples was so small that it could not be detected by visual observation. The watermelon rind powder was slightly darker than the durum wheat semolina. Different colouring compounds such as carotenoids and anthocyanins were identified in durum wheat semolina (*40*) and watermelon rind (*10*). In addition, reducing sugars were found in the watermelon rind powder. It can be concluded that a Maillard reaction between reducing sugars and proteins could take place during the drying of watermelon rind at 50 °C (*41*), resulting in an increased darkness of the watermelon rind powder.

The WHC of watermelon rind powder was much higher than that of durum wheat semolina. Dietary fibre has a high affinity for water because of the hydroxyl groups (*42*). The smaller the mean particle size of the watermelon rind powder sample, the lower the WHC due to the lower total fibre. In addition, the watermelon rind sample with reduced particle size was rich in pectin, which can be partially lost in the aqueous phase during the determination of WHC (*2*). The reduction in WHC of pomelo peel powder sample with the reduced particle size was reported previously (*43*). Swelling of fibre materials is affected by their water absorption (*42*) and the watermelon rind powder sample with smaller particle size had lower swelling capacity. However, the WSI of watermelon rind powder gradually increased with the reduction of particle size because of the higher content of SDF (*42*).

### Effect of the particle size of watermelon rind powder on nutritional quality of pasta

Table 2 shows the nutritional quality of pasta samples. All pasta samples with added watermelon rind had much more dietary fibre than the control sample. The reduced particle size of the watermelon rind powder increased SDF and pectin content of the pasta, but decreased its TDF and IDF content. According to the Codex Alimentarius (2013) Guidelines for Use of Nutrition and Health Claims (*44*), all pasta samples containing watermelon rind in this study were classified as high-fibre foods since their TDF content on dry mass basis was above 6 g/100 g. In addition, the IDF/SDF ratio of the high-fibre pasta samples progressively decreased when the sieve aperture size for watermelon rind powder was reduced from 400 to 149 µm. It can be concluded that all pasta samples supplemented with watermelon rind powder had an IDF/SDF ratio suitable for human consumption, since the ratio was almost within the range of [1-3] recommended by the American Dietetic Association (*3*, *29*). A high IDF/SDF ratio is usually observed in pasta fortified with cereal bran such as wheat bran (7.0) (*3*) or rice bran (9.0) (*31*). The use of watermelon rind powder in pasta formulation also increased the ash content but decreased the starch content. Nevertheless, the protein content of the high-fibre pasta samples and the control sample were statistically similar. The change in the nutritional quality of the pasta samples was due to the different proximate composition of durum wheat semolina and watermelon rind powder.

**Table 2:** Nutritional quality on dry mass basis of raw pasta samples

Parameter	P400	P210	P149	Control
*w*(water)/%	(10.0±0.1)ᵃ	(10.0±0.1)ᵃ	(10.1±0.1)ᵃ	(12.3±0.1)ᵇ
*w*(TDF)/(g/100 g)	(7.5±0.1)ᵈ	(7.3±0.1)ᶜ	(6.9±0.2)ᵇ	(3.6±0.1)ᵃ
*w*(IDF)/(g/100 g)	(5.7±0.1)ᵈ	(5.3±0.1)ᶜ	(4.8±0.2)ᵇ	(2.9±0.1)ᵃ
*w*(SDF)/(g/100 g)	(1.8±0.1)ᵇ	(2.0±0.1)ᶜ	(2.1±0.1)ᵈ	(0.7±0.0)ᵃ
*w*(pectin)/(g/100 g)	(1.0±0.1)ᵃ	(1.2±0.1)ᵇ	(1.4±0.1)ᶜ	n.d.
*ζ*(IDF/SDF)/(g/g)	(3.2±0.2)ᶜ	(2.6±0.1)ᵇ	(2.3±0.2)ᵃ	(4.0±0.2)ᵈ
*w*(starch)/(g/100 g)	(63.5±2.1)ᵃ	(63.4±0.8)ᵃ	(64.4±1.1)ᵃ	(71.2±1.7)ᵇ
*w*(protein)/(g/100 g)	(13.3±0.5)ᵃ	(13.4±0.4)ᵃ	(13.5±0.3)ᵃ	(13.0±0.5)ᵃ
*w*(lipid)/(g/100 g)	(2.1±0.1)ᵇ	(2.1±0.1)ᵇ	(2.0±0.1)ᵇ	(1.8±0.1)ᵃ
*w*(ash)/(g/100 g)	(1.6±0.1)ᵇ	(1.6±0.0)ᵇ	(1.6±0.1)ᵇ	(0.8±0.0)ᵃ

Values with different lowercase letters in the same row are significantly different (p<0.05). n.d.=not detected, TDF=total dietary fibre, IDF=insoluble dietary fibre, SDF=soluble dietary fibre, P400, P210 and P149=pasta with 10 % watermelon rind powder sifted through *d*(aperture sieve)=400, 210 and 149 μm, respectively

### Effect of the particle size of watermelon rind powder on in vitro bioaccessibility of phenols, antioxidant activity and predicted glycaemic index of pasta

**Fig. 1:**
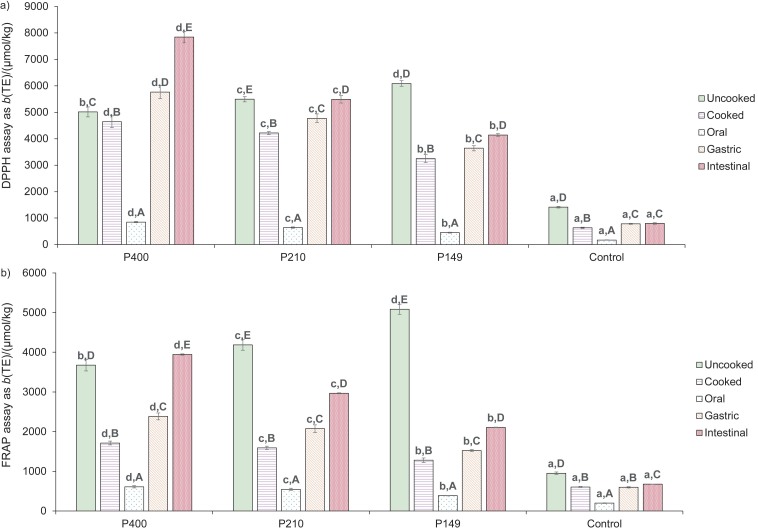
Antioxidant activity on dry mass basis of pasta sample according to: a) DPPH, and b) FRAP assays. Values with different lowercase letters in the same legend and uppercase in the same sample are significantly different (p<0.05). TE=Trolox equivalent, FRAP=Fe(II) reducing antioxidant power, DPPH=2,2-diphenyl-1-picrylhydrazyl radical scavenging activity, P400, P210 and P149=pasta with 10 % watermelon rind powder passed through *d*(aperture sieve)=400, 210 and 149 μm, respectively

The antioxidant content and activities of all pasta samples are shown in Table 3 and Fig. 1, respectively. The addition of watermelon rind powder to pasta recipe greatly improved total phenolic, flavonoid and anthocyanin mass fractions of the raw product. When the sieve opening size was reduced from 400 to 149 μm, the raw pasta containing watermelon rind had lower mass fraction of anthocyanins, while its TPC and TFC as well as antioxidant activities were significantly improved. That was due to the difference in phenolic mass fraction of watermelon rind powder samples.

**Table 3:** Bioactive compounds determined on dry mass basis of pasta samples

Parameter			Raw pasta	Cooked pasta	Cooked pasta during the *in vitro* digestion		
Oral	Gastric	Intestinal
Total phenolics as *w*(GAE)/(mg/kg)		P400	(1291±35)^b,C^	(1023±50)^d,B^	(200±8)^d,A^	(1253±35)^d,C^	(1650±44)^d,D^
		P210	(1457±72)^c,D^	(769±35)^c,B^	(137±4)^c,A^	(1013±47)^c,B^	(1172±44)^c,C^
		P149	(1668±72)^d,E^	(614±28)^b,B^	(84±0)^b,A^	(654±17)^b,C^	(849±3)^b,D^
		Control	(353±13)^a,D^	(292±6)^a,C^	(49±0)^a,A^	(252±11)^a,B^	(269±8)^a,B^
Total flavonoids as *w*(QE)/(mg/kg)		P400	(800±28)^a,B^	(655±15)^c,A^	n.d.	(789±13)^c,B^	(1038±39)^c,C^
		P210	(857±26)^b,D^	(462±22)^b,A^	n.d.	(666±22)^b,B^	(744±16)^b,C^
		P149	(978±39)^c,D^	(371±12)^a,A^	n.d.	(432±18)^a,B^	(560±17)^a,C^
		Control	n.d.	n.d.	n.d.	n.d.	n.d.
Total anthocyanin as *w*(CGE)/(mg/kg)		P400	(74±2)^c,B^	(56±2)^c,A^	n.d.	(83±1)^c,C^	(95±3)^c,D^
		P210	(70±2)^b,C^	(36±1)^b,A^	n.d.	(51±2)^b,B^	(55±2)^b,B^
		P149	(65±3)^a,D^	(22±1)^a,A^	n.d.	(28±1)^a,B^	(36±1)^a,C^
		Control	n.d.	n.d.	n.d.	n.d.	n.d.
Correlation value (r)	TPC and antioxidant activity based on DPPH assay		0.998	0.929	0.978	0.959	0.992
	TPC and antioxidant activity based on FRAP assay		0.998	0.942	0.932	0.966	0.991
	DPPH scavenging activity and FRAP assay		0.992	0.999	0.987	0.997	0.998

Values with different uppercase letters in the same row and lowercase letters in the same column are significantly different (p<0.05). n.d.=not detected, GAE=gallic acid equivalent, QE=quercetin equivalent, CGE=cyanidin-3-glucoside equivalent, TPC=total phenolic content, FRAP=Fe(III) reducing antioxidant power, DPPH=2,2-diphenyl-1-picrylhydrazyl radical scavenging activity, P400, P210 and P149=pasta with 10 % watermelon rind powder sifted through *d*(aperture sieve)=400, 210 and 149 μm, respectively

The cooking reduced the TPC, TFC and TAC and antioxidant activity of all pasta samples. Phenols are reported to be partially lost during pasta cooking due to oxidative degradation (*45*). The smaller the watermelon rind particle size, the higher the loss of phenols and antioxidant activity. This could be explained by the longer optimal cooking time of pasta fortified with watermelon rind powder with finer particles. In addition, the amount of phenols and the DPPH scavenging activity of the cooked pasta supplemented with watermelon rind powder samples, expressed on dry mass basis as gallic acid and Trolox equivalents, were 614 to 1023 mg/kg and 3252 to 4642 μmol/kg, respectively, higher than those of the cooked control sample.

**Fig. 2:**
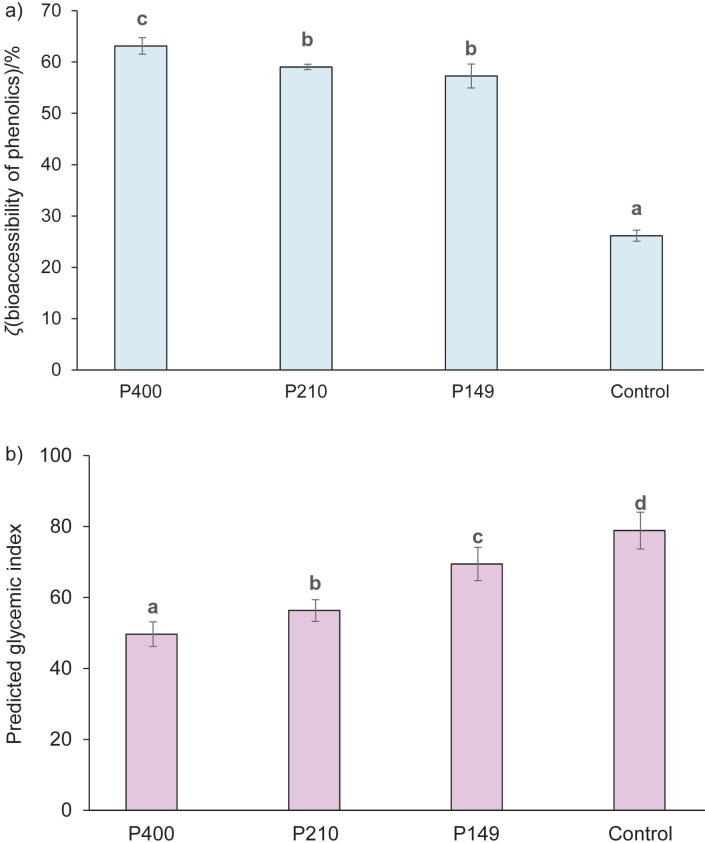
Bioaccessibility of total phenols (a), and predicted glycaemic index (b) of pasta samples. Values with different lowercase letters in the same chart are significantly different (p<0.05). P400, P210 and P149=pasta incorporated with 10 % watermelon rind powder passed through *d*(aperture sieve)=400, 210 and 149 μm, respectively

In the oral phase of the *in vitro* digestion, the TPC as well as the DPPH scavenging activity and Fe(III) reducing power were lowest in all pasta samples, while flavonoids and anthocyanins could not be detected. This is probably due to the short exposure time to salivary amylase (2 min). Recent *in vitro* digestion shows that different flavonoids such as catechin and epicatechin in pasta supplemented with persimmon flour are not released during oral digestion since they bind various organic compounds (*28*). The release of antioxidant content and activity from the control and pasta samples with watermelon rind was greatly improved in the gastric phase; flavonoids and anthocyanins were observed in the gastric juice. This can be explained by the acidic degradation of condensed tannin under low pH conditions (*28*) and proteolysis by pepsin (*46*, *47*), which facilitates the release of bound phenolic molecules (*28*). In the intestinal phase, both the TPC and the antioxidant activity of the control pasta remained almost unchanged compared to those in the gastric phase. Nevertheless, the TPC, TFC and TAC as well as DPPH radical scavenging activity and Fe(III) reducing power released from the pasta samples supplemented with watermelon rind all reached maximum values. The main reason for this is probably the hydrolysis of starch, lipids and proteins at pH=7.0 by amylase, lipase, trypsin and other proteases in the simulated intestinal fluid (*47*); this enhanced the release of bound phenols from starch, lipid and protein matrix. Similar increase in TPC and antioxidant activity was reported by Bustos *et al.* (*46*) when pasta prepared with berry fruits was used in the simulated digestion model. However, a lower TAC was reported in the intestinal phase than in the gastric phase when the peel of jaboticaba (*Myrciaria trunciflora*) fruit was used in the *in vitro* gastrointestinal digestion (*48*). Different results are due to the difference in phenolic profile and the interactions between phenols and organic compounds in the plant materials. A detailed study on the release and stability of individual phenolic compounds from the pasta with added watermelon rind at different digestion phases is essential to clarify the effect of antioxidants in high-fibre pasta on human health. It can be noted that the larger the particle size of watermelon rind powder, the more the antioxidant contents and activities released from the fortified pasta in the oral, gastric and intestinal phases of the *in vitro* digestion. It has been reported that many phenolic compounds in watermelon rind bind to dietary fibre in the plant cell wall (*49*) and could be released during human digestion (*29*). The bioaccessibility of total phenols and predicted glycaemic index of all pasta samples are shown in Fig 2.

Fig. 2a shows that the bioaccessibility of total phenols in the pasta with watermelon powder was much higher than that of the control pasta. The pasta containing watermelon rind powder with the largest mean particle size had the highest phenolic bioaccessibility. The glycaemic index (GI) (Fig. 2b) is an indicator of how much a carbohydrate-rich food increases blood sugar level in the human body. The control pasta sample had the highest pGI since it had the highest starch content. Although the three pasta samples with watermelon rind had similar starch content, the increased pGI was observed in the sample supplemented with watermelon rind powder with reduced mean particle size. This can be explained by the reduced TDF content. Insoluble fibre-rich fractions reduced the swelling of starch granules in cooked pasta due to their high water absorption, resulting in the limited starch hydrolysis (*3*, *50*). Furthermore, the interaction of dietary fibre with starch (*50*), as well as the complex formation of soluble fibre (*3*) and polyphenols (*50*) with α-amylase, could limit the accessibility of amylase to starch granules in pasta. The P400 sample was considered a low pGI product (pGI<55), while the other pasta samples with watermelon rind were in the medium pGI category (55<pGI<70). The control pasta sample was considered a high pGI product (*51*). The diet with low pGI products may reduce the risk of obesity, diabetes and cardiovascular disease (*3*). It can be confirmed that the addition of watermelon rind powder to the pasta recipe significantly reduced the predicted glycaemic index of the product.

### Effect of the particle size of watermelon rind powder on texture quality and colour of pasta

Table 4 shows textural attributes of the cooked pasta samples and colour values of the raw samples.

**Table 4:** Textural attributes of cooked pasta and instrumental colour values of raw pasta samples

Parameter	P400	P210	P149	Control
Hardness/g	(2664±106)ᵈ	(2463±109)ᶜ	(2328±105)ᵇ	(2008±56)ᵃ
Adhesiveness	(14.68±0.36)ᵈ	(13.03±0.30)ᶜ	(12.23±0.34)ᵇ	(10.64±0.50)ᵃ
Tensile strength/kPa	(22.2±0.7)ᵃ	(23.5±0.6)ᵇ	(25.0±0.8)ᶜ	(27.1±0.6)ᵈ
Elongation rate/%	(32.3±1.2)ᵃ	(41.1±1.2)ᵇ	(45.2±3.1)ᶜ	(57.3±1.1)ᵈ
L*	(87.4±0.1)ᵃ	(87.6±0.2)ᵇ	(87.8±0.0)ᶜ	(89.3±0.0)ᵈ
a*	(1.2±0.0)ᵇ	(1.4±0.1)ᶜ	(1.4±0.0)ᶜ	(1.1±0.0)ᵃ
b*	(11.8±0.1)ᵇ	(12.4±0.0)ᶜ	(12.8±0.1)ᵈ	(8.7±0.0)ᵃ
Δ*E*	(3.6±0.1)ᵇ	(4.0±0.1)ᶜ	(4.3±0.0)ᵈ	n.a.

Values with different lowercase letters in the same chart are significantly different (p<0.05). n.a.=not application, P400, P210 and P149=pasta with 10 % watermelon rind powder sifted through *d*(aperture sieve)=400, 210 and 149 μm, respectively

The use of watermelon rind powder in the pasta formulation increased the product hardness. It is possible that the high degree of polymerization and crystallization of IDF of WR powder (*52*) could increase the hardness of the pasta with watermelon rind (*53*). When the sieve aperture size was reduced from 400 to 149 μm, the hardness of the pasta samples gradually decreased probably due to the lower IDF content. Adhesiveness shows the force required to overcome the attractive forces between the food surface and the probe surface with which the food comes into contact. The control pasta sample had the lowest adhesiveness. The adhesiveness of the fibre-rich pasta increased approximately when the particle size of the watermelon rind powder was increased. This may be attributed to fibre components that disrupt the protein-starch network and result in a greater release of exudates from pasta strands into cooking water, leading to the increased adhesiveness (*54*). During our experiments, we observed that the higher the pasta fibre content, the higher the number of water droplets on the pasta strand surface after the same draining time. The use of watermelon rind powder in pasta formulation significantly reduced the elongation rate and tensile strength of the pasta since the reduced gluten content and the presence of dietary fibre in the gluten network of the pasta dough could reduce its continuity (*3*, *55*). Moreover, fibre could alter the β‐sheet structure of gluten, leading to protein folding and preventing the formation of disulfide bonds for gluten linkage (*56*, *57*), which would result in the reduced tensile strength. The improved elongation rate and tensile strength of high-fibre pasta were accompanied by a decrease in watermelon rind particle size.

**Fig. 3:**
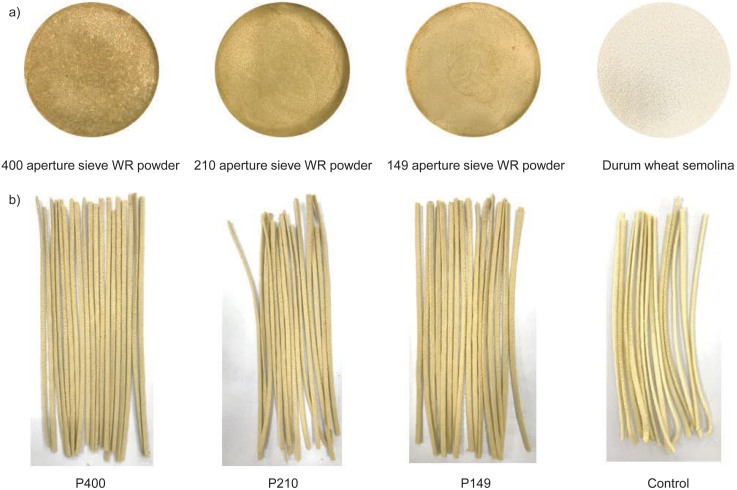
Photographs of: a) watermelon rind (WR) powder and durum wheat semolina, and b) raw pasta fortified with watermelon rind and control pasta. P400, P210 and P149=pasta with 10 % watermelon rind powder passed through *d*(aperture sieve)=400, 210 and 149 μm, respectively

Fig. 3a and Fig. 3b show the photographs of material and raw pasta samples, respectively. The lightness of the pasta samples with added watermelon rind was slightly lower than that of the control pasta (Fig. 3b) since the watermelon rind powder was darker than the durum wheat semolina (Fig. 3a). The change in the mean watermelon rind particle size had a little effect on the colour of the high-fibre pasta samples and these changes were not perceptible to the eye.

### Effect of the particle size of watermelon rind powder on cooking quality and overall sensory acceptability of pasta

Table 5 shows that the OCT for high fibre-pasta samples was shorter while the CL was significantly higher than that of the control pasta, probably due to a reduced gluten content (*3*, *54*). Low OCT could save more cooking energy and limit the loss of bioactive compounds. It can be noted that the CL of all pasta samples with added watermelon rind was lower than the acceptable limit (8 %) (*9*). When the sieve aperture size for watermelon rind powder was reduced from 400 to 149 µm, the OCT of the high-fibre pasta was steadily prolonged while its CL decreased by 15 %, probably due to a better distribution of watermelon rind particles in the pasta dough since the obtained watermelon rind powder sample had a reduced span value (Table 1), resulting in the improved gluten network. Similar decrease in the CL was also observed when the potato peel powder with fine particles was added to pasta formulation (*35*). In addition, watermelon rind powder with large particle size could disrupt gluten network even more, making pasta with a less uniform texture, accelerating starch gelatinization through increased diffusion of cooking water (*55*).

**Table 5:** Cooking quality and overall acceptability of cooked pasta samples

Parameter	P400	P210	P149	Control
Optimal cooking time/min	(10.8±0.3)ᵃ	(11.9±0.2)ᵇ	(12.7±0.3)ᶜ	(13.9±0.2)ᵈ
Cooking loss/%	(6.6±0.3)ᵈ	(6.1±0.2)ᶜ	(5.6±0.2)ᵇ	(4.6±0.1)ᵃ
Swelling index	(1.67±0.02)ᵃ	(1.69±0.00)ᵃ	(1.69±0.08)ᵃ	(1.83±0.05)ᵇ
Water absorption index	(1.22±0.02)ᵃ	(1.29±0.04)ᵇ	(1.40±0.03)ᶜ	(1.56±0.04)ᵈ
Overall acceptability	(6.3±1.0)ᵃ	(6.2±1.0)ᵃ	(5.9±1.1)ᵃ	(6.3±1.1)ᵃ

Values with different lowercase letters in the same row are significantly different (p<0.05). P400, P210 and P149=pasta with 10 % watermelon rind powder sifted through *d*(aperture sieve)=400, 210 and 149 μm, respectively

Change in the particle size of the watermelon rind powder did not alter the swelling index of high-fibre pasta samples but it was lower than that of the control, possibly due to the reduction in starch content (*53*). Similarly, the WAI of the control pasta was slightly higher than that of the high fibre pasta. The use of watermelon rind powder with reduced particle size increased the WAI of high-fibre pasta because of prolonged OCT, which improved water absorption of starch, fibre and protein during cooking (*53*). The overall sensory acceptability was statistically equivalent for all pasta samples. The change in the particle size of watermelon rind powder did not affect the acceptance level of the pasta.

## CONCLUSIONS

The particle size of the watermelon rind powder, whether large or small, had different effects on the quality of high-fibre pasta. The use of watermelon rind powder with larger particle size gave pasta with increased dietary fibre content, improved antioxidant activity and bioaccessibility, and reduced glycaemic index. At the same time, the texture, colour and cooking quality of the functionalized pasta were reduced. However, there was no discernible difference in overall acceptability between the control pasta sample and the three high-fibre pasta samples with added watermelon rind powder that were sifted through sieves with the aperture of 400, 210 and 149-μm. In the future, the bioaccessibility of individual phenolic compounds in the pasta supplemented with watermelon rind should be investigated at each digestion step for a better understanding of phenolic metabolism in human nutrition. Furthermore, the effect of particle size of dietary fibre material in functional pasta on the gut microbiota and colonic proliferation should be investigated.
